# Supplementary figures and images for: Biofilm dispersal patterns revealed using far-red fluorogenic probes
Source: PLoS Biol. 2024 Nov 25;22(11):e3002928. doi: 10.1371/journal.pbio.3002928 (PMC11627390; doi:10.1371/journal.pbio.3002928)

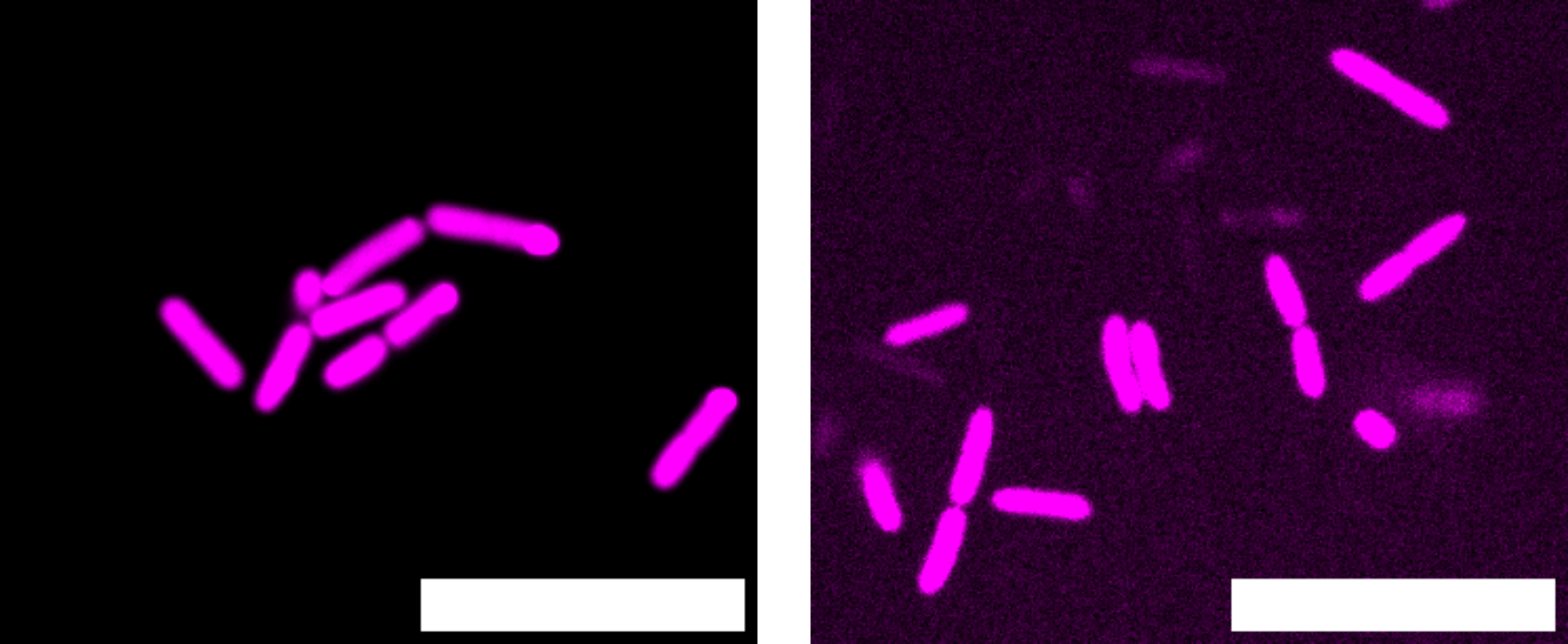

Supplement: S1 Fig — (A) E. coli. (B) P. aeruginosa PA14. Scale bar is 10 μm. (TIFF) [file pbio.3002928.s001.tiff]

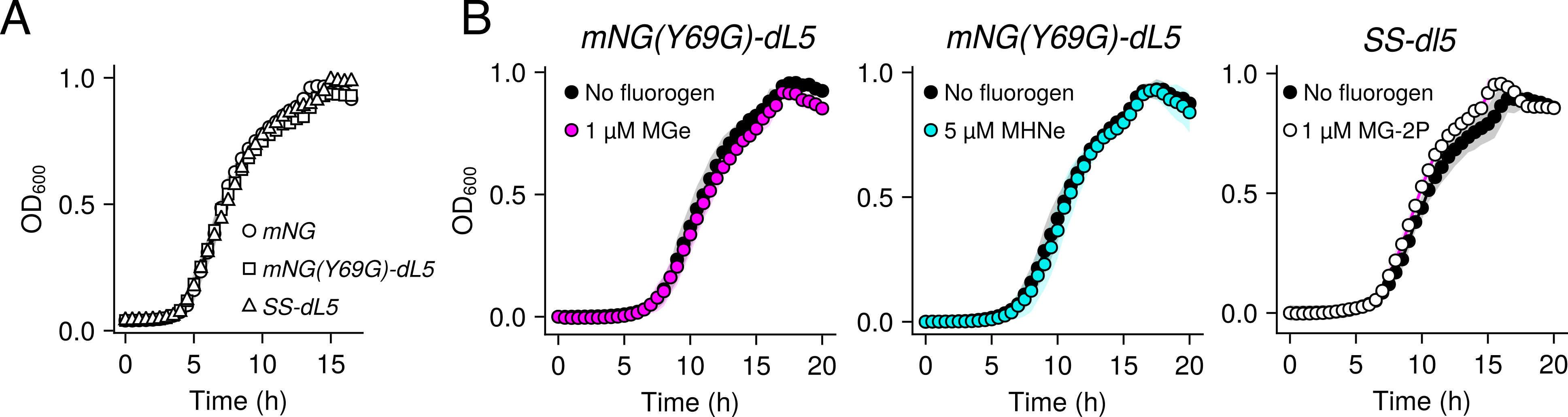

Supplement: S2 Fig — (A) Growth curves at 30°C for a strain constitutively expressing the cytoplasmic fluorescent protein mNeonGreen (Ptac-mNeonGreen) compared to strains constitutively expressing dL5 targeted to the cytoplasm (Ptac-mNeonGreen(Y69G)-dL5) or the periplasm (Ptac-SS-dL5), with no fluorogen added. (B) Growth curves for the indicated strains grown in the absence or presence of their cognate fluorogens at the indicated concentrations. In all cases, N = 3 biological replicates. Points represent means and shading represents standard deviations. Underlying data for this figure can be found on Figshare (https://figshare.com/s/e0978ade2bc95dccf357). (TIFF) [file pbio.3002928.s002.tiff]

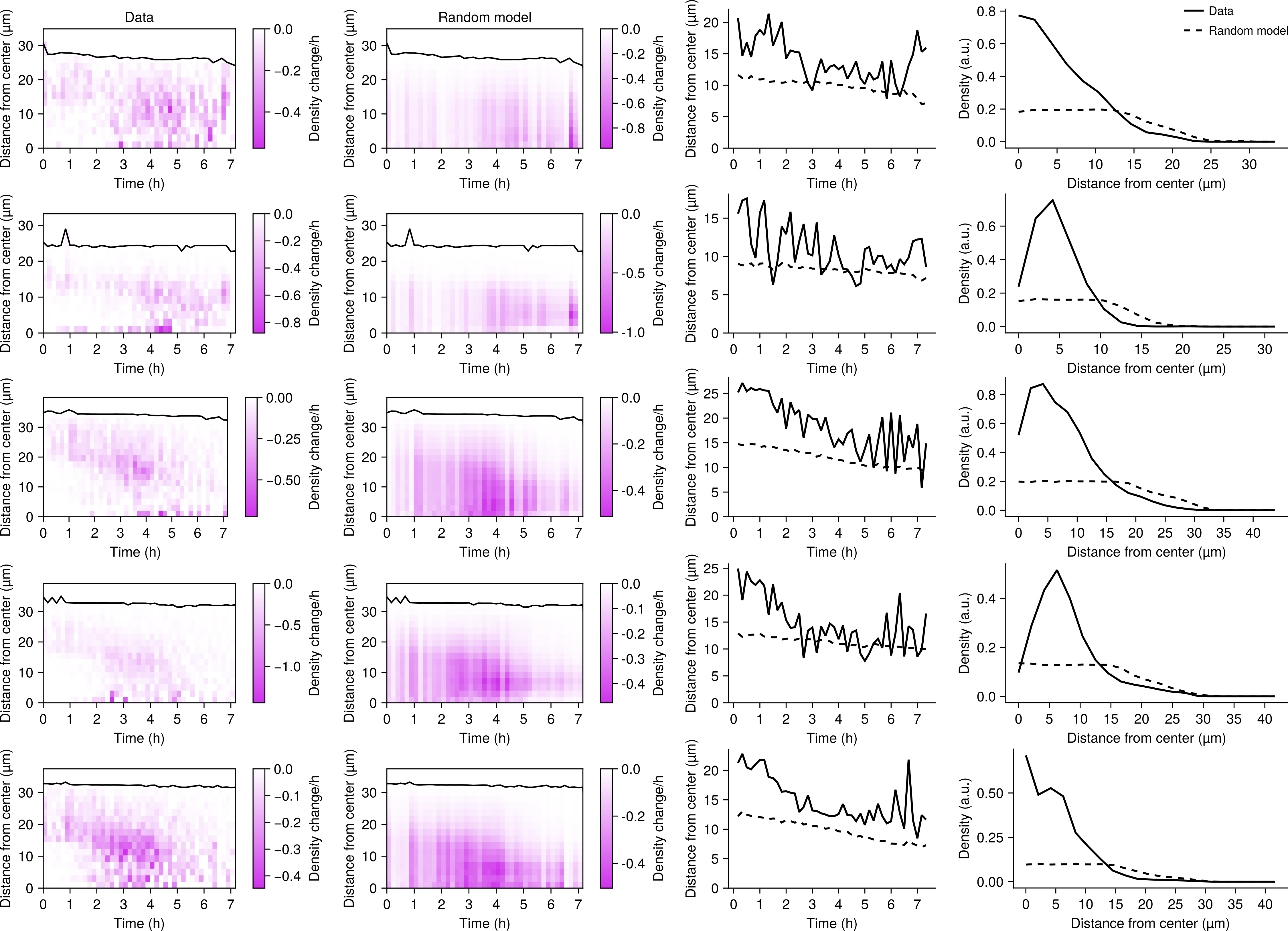

Supplement: S4 Fig — Black line represents the biofilm boundary. Each panel represents data from a single biofilm for N = 5 biofilms. Second column from the left: as in the left-most column for the “Random” model. The rate of overall cell departure was set to match the experimental data for each biofilm. Second column from the right: centroid position of the density change data in the left panels over time. Right-most column: spatial distribution of the local density profiles for the data and random model at the completion of dispersal. a.u., arbitrary units. Underlying data for this figure can be found on Figshare (https://figshare.com/s/e0978ade2bc95dccf357). (TIFF) [file pbio.3002928.s004.tiff]

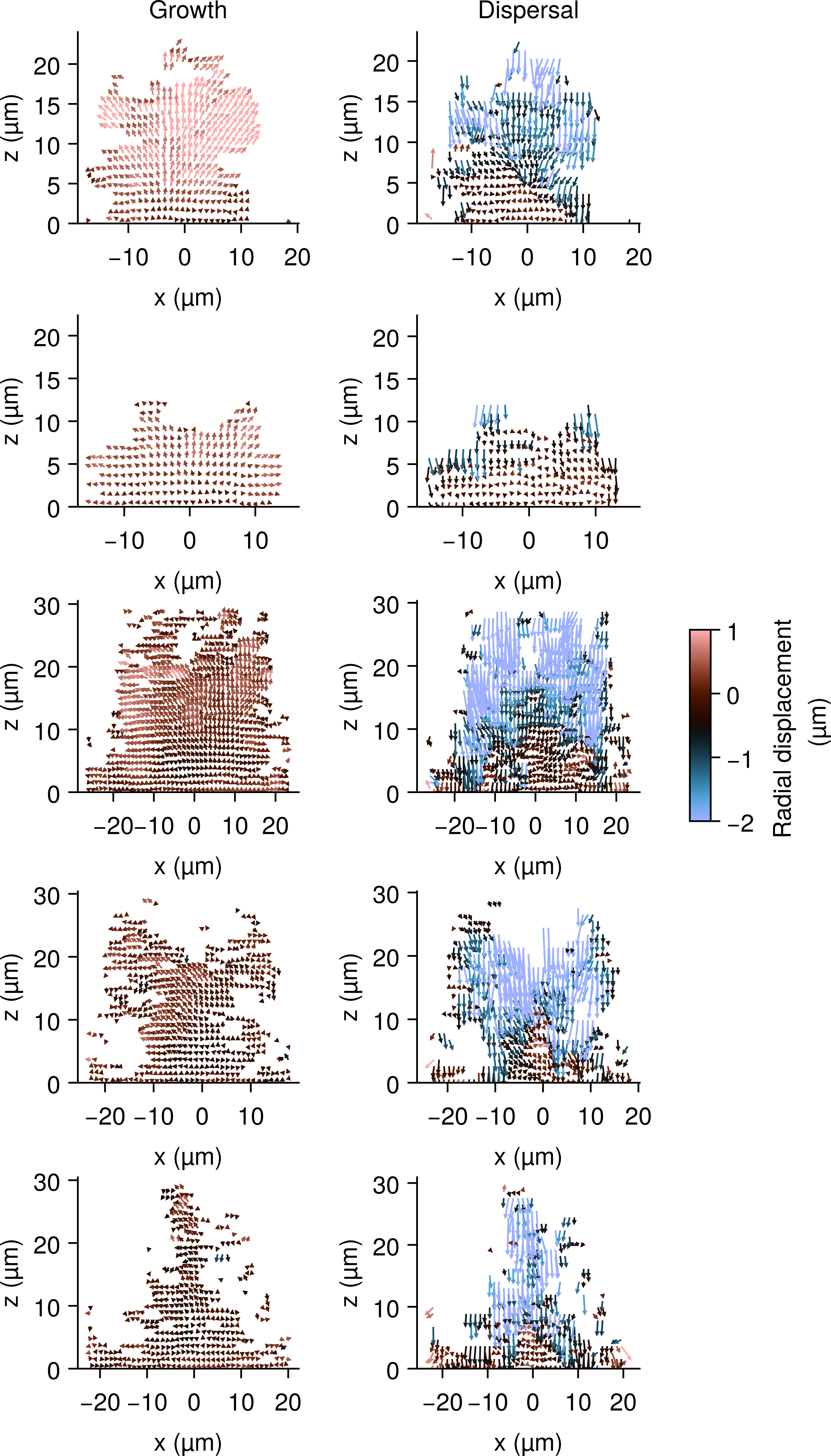

Supplement: S5 Fig — Each pair of “Growth” and “Dispersal” panels represents the displacement vectors during a ~1.5-h period preceding dispersal (“Growth”) and throughout dispersal (“Dispersal”) for an individual wild-type biofilm. Vector color represents the radial displacement with respect to the core of the biofilm. For ease of demonstration, a single slice through the y-axis is shown. Colorbar is the same for all panels. Underlying data for this figure can be found on Figshare (https://figshare.com/s/e0978ade2bc95dccf357). (TIFF) [file pbio.3002928.s005.tiff]
