# Supplementary material for: Biofilm dispersal patterns revealed using far-red fluorogenic probes
Source: PLoS Biol. 2024 Nov 25;22(11):e3002928. doi: 10.1371/journal.pbio.3002928 (PMC11627390; doi:10.1371/journal.pbio.3002928)
Supplement: S1 Text — Measurement of binding properties of FAPs expressed in V. cholerae. (DOCX) [file pbio.3002928.s016.docx]

**S1 Text: FAP labeling equilibria and kinetics**

The addition of the fluorogens used here had no effect on the growth rate of *V.* *cholerae* at the highest concentrations tested (S2 Fig), and the measured EC_50_ was lower for MGe and MG-2P (0.1 µM) than for MHNe (0.6 µM), reflecting differences in dL5 affinities (S3A Fig). Moreover, the fold change in constitutive bulk culture fluorescence relative to background was highest in the MG-2P treated cultures (182x above background), followed by cytoplasmic labeling with MGe (~90x), and finally MHNe (~2.9x) (S3B Fig). Generally, we found it most useful to include fluorogens in media throughout growth; however, to evaluate the dynamics of fluorogen labeling, we pre-grew large biofilms for 20 hours using norspermidine, removed planktonic cells and exogenously added saturating concentrations of MG-2P and MGe. MG-2P and MGe readily penetrated pre-existing biofilms, reaching equilibrium at ~45 minutes, and 90 minutes post-addition, respectively (S3C Fig). Given the superior fluorescence and faster binding kinetics of the periplasmic SS-dL5/MG2-P labeling approach, we exclusively used this approach to investigate biofilm dynamics at high spatiotemporal resolution.
