# Supplementary material for: Biofilm dispersal patterns revealed using far-red fluorogenic probes
Source: PLoS Biol. 2024 Nov 25;22(11):e3002928. doi: 10.1371/journal.pbio.3002928 (PMC11627390; doi:10.1371/journal.pbio.3002928)
Supplement: S1 Table — List of strains used in the study. (DOCX) [file pbio.3002928.s017.docx]

**S1 Table. Strains used in this study.**

| Strain number | Genotype | Plasmid | Antibiotic resistance | Origin |
| --- | --- | --- | --- | --- |
| AB_Vc_707 | Vibrio cholerae C6706str2 | - | - | - |
| AB_Vc_761 | ∆vc1807::cm^R^ | - | Cm^R^ | NT of AB_Vc_707 |
| RW_VC_1545 | Ptac-mNeonGreen-dL5 ∆vc1807::kan^R^ | - | Kan^R^ | NT of AB_Vc_707 |
| AB_VC_1717 | Ptac- mNeonGreen (Y69G)-dL5 ∆vc1807::kan^R^ | - | Kan^R^ | NT of AB_Vc_707 |
| RW_Vc_1757 | Ptac-dL5-µNS ∆vc1807::kan^R^ | - | Kan^R^ | NT of AB_Vc_707 |
| RW_Vc_1892 | Ptac-ss(MBP)-dL5  ∆vc1807:: spec^R^ | - | Spec^R^ | NT of AB_Vc_707 |
| AB_Ec_116 | E. coli BW25113 | pBBR1::Ptac-mNeonGreen-dL5 | Gent^R^ | Trans of AB_Ec_099 |
| AB_NV_026 | P.aeruginosa PA14 | pBBR1::Ptac-mNeonGreen-dL5 | Gent^R^ | Trans of AB_NV_005 |
| RW_Vc_1675 | ∆cheY3 Ptac-ss(MBP)-dL5  ∆vc1807::kan^R^ | - | Kan^R^ | NT of AB_Vc_705 |
| RW_Vc_1673 | ∆lapG Ptac-ss(MBP)-dL5  ∆vc1807::kan^R^ | - | Kan^R^ | NT of AB_Vc_757 |
| RW_Vc_1677 | ∆rbmB Ptac-ss(MBP)-dL5  ∆vc1807:: kan^R^ | - | Kan^R^ | NT of AB_Vc_182 |
| JP_Vc_1970 | ∆rbmA Ptac-ss(MBP)-dL5  ∆vc1807:: spec^R^ | - | Spec^R^ | NT of AB_Vc_1427 |

NT; natural transformation. Trans; transformation.
