# Supplementary material for: Biofilm dispersal patterns revealed using far-red fluorogenic probes
Source: PLoS Biol. 2024 Nov 25;22(11):e3002928. doi: 10.1371/journal.pbio.3002928 (PMC11627390; doi:10.1371/journal.pbio.3002928)
Supplement: S2 Table — Color coded DNA sequences for FAP expression. (DOCX) [file pbio.3002928.s018.docx]

**S2 Table. FAP-fusion sequences used in this study.**

1. ***Ptac-mNeonGreen-dL5***

***promoter-mNeonGreen-linker-dL5***

TGCACCAATGCTTCTGGCGTCAGGCAGCCATCGGAAGCTGTGGTATGGCTGTGCAGGTCGTAAATCACTGCATAATTCGTGTCGCTCAAGGCGCACTCCCGTTCTGGATAATGTTTTTTGCGCCGACATCATAACGGTTCTGGCAAATATTCTGAAATGAGCTGTTGACAATTAATCATCGGCTCGTATAATGTGTGGAATTGTGAGCGGATAACAATTTCACACAGGAAACAGCCTCGACAGGCCTAGGAATTCAATTAGGAGGTAATTAAGCATGGTATCGAAGGGGGAGGAAGATAATATGGCTTCACTTCCAGCTACACACGAGCTTCACATTTTTGGTTCTATCAACGGAGTTGACTTTGACATGGTCGGACAAGGTACTGGCAATCCGAACGACGGTTACGAGGAATTAAATCTTAAATCTACGAAAGGGGACTTACAGTTTTCACCCTGGATCTTAGTGCCGCATATCGGTTACGGGTTCCACCAATATCTCCCGTACCCTGACGGAATGTCGCCTTTCCAGGCAGCTATGGTGGACGGTTCAGGGTATCAGGTACATCGTACCATGCAATTTGAAGATGGCGCATCTCTTACAGTGAACTATCGCTACACGTATGAAGGTAGTCACATCAAGGGTGAGGCCCAAGTAAAGGGGACAGGCTTCCCAGCTGACGGACCTGTTATGACAAATTCGTTGACAGCGGCAGATTGGTGCCGCTCTAAAAAGACTTACCCTAATGACAAGACTATTATCAGTACCTTCAAGTGGAGCTACACAACGGGTAACGGGAAACGCTACCGTTCAACGGCACGCACCACCTATACTTTCGCAAAACCGATGGCAGCCAACTACCTCAAGAATCAACCAATGTATGTATTCCGCAAAACCGAACTGAAGCACTCGAAGACCGAACTGAACTTTAAGGAATGGCAAAAAGCCTTTACAGACGTTATGGGGATGGATGAATTATATAAGGGCGGTGGGGGCTTGTCTGCTGGCGCTGGGGGCGGAGGCAGCGCGTCTCAGGCCGTAGTGACCCAAGAACCCAGTGTGACTGTCAGTCCAGGAGGCACCGTGATCTTAACTTGTGGTTCAGGTACTGGGGCGGTTACCAGCGGTCATTATGCTAATTGGTTTCAGCAAAAGCCAGGACAAGCTCCGCGCGCGCTTATTTTCGACACCGACAAGAAATACTCTTGGACTCCTGGGCGCTTTTCAGGCAGTTTACTGGGCGCGAAAGCGGCTTTGACGATCTCTGACGCACAGCCAGAGGATGAAGCGGAATATTACTGTAGCCTGTCTGACGTAGATGGCTATTTATTTGGCGGAGGTACTCAACTTACGGTGTTGTCCGGAGGCGGTGGGTCGGGCGGTGGGGGCTCCGGAGGTGGCGGAAGCGGAGGGGGAGGCAGCCAAGCAGTGGTTACTCAGGAGCCGTCTGTTACCGTGTCACCGGGCGGTACTGTGATTCTGACCTGCGGCTCGGGAACTGGGGCGGTCACCTCAGGGCACTATGCAAATTGGTTTCAACAGAAACCCGGTCAAGCGCCTCGCGCACTTATCTTTGATACTGATAAGAAATATAGTTGGACGCCAGGTCGTTTCAGCGGTTCTCTGTTGGGCGCAAAAGCTGCGCTTACCATTAGCGATGCGCAACCTGAGGACGAGGCCGAGTACTATTGTTCGCTGTCGGATGTAGATGGATACCTATTTGGTGGCGGAACACAACTAACGGTGCTGTCCACCGGGCATCACCATCACCATCACTAA

1. ***Ptac-SS-dL5***

***promoter -secretion signal (MBP)-linker-dL5***

TGCACCAATGCTTCTGGCGTCAGGCAGCCATCGGAAGCTGTGGTATGGCTGTGCAGGTCGTAAATCACTGCATAATTCGTGTCGCTCAAGGCGCACTCCCGTTCTGGATAATGTTTTTTGCGCCGACATCATAACGGTTCTGGCAAATATTCTGAAATGAGCTGTTGACAATTAATCATCGGCTCGTATAATGTGTGGAATTGTGAGCGGATAACAATTTCACACAGGAAACAGCCTCGACAGGCCTAGGAATTCAATTAGGAGGTAATTAAGCTTGGTGAGCCCTACAACACAAAAAGAAAAGGATATGAACATGAAAAATGCCCTAAGCACAGTCGCGCTGAGCACTCTGGTGGCTCTTGGTTCGTTTGGTGCCCATGCTGCTGAAGCGGAAGCATCTGCTGGCGCTGGGGGCGGAGGCAGCGCGTCTATGCAGGCCGTAGTGACCCAAGAACCCAGTGTGACTGTCAGTCCAGGAGGCACCGTGATCTTAACTTGTGGTTCAGGTACTGGGGCGGTTACCAGCGGTCATTATGCTAATTGGTTTCAGCAAAAGCCAGGACAAGCTCCGCGCGCGCTTATTTTCGACACCGACAAGAAATACTCTTGGACTCCTGGGCGCTTTTCAGGCAGTTTACTGGGCGCGAAAGCGGCTTTGACGATCTCTGACGCACAGCCAGAGGATGAAGCGGAATATTACTGTAGCCTGTCTGACGTAGATGGCTATTTATTTGGCGGAGGTACTCAACTTACGGTGTTGTCCGGAGGCGGTGGGTCGGGCGGTGGGGGCTCCGGAGGTGGCGGAAGCGGAGGGGGAGGCAGCCAAGCAGTGGTTACTCAGGAGCCGTCTGTTACCGTGTCACCGGGCGGTACTGTGATTCTGACCTGCGGCTCGGGAACTGGGGCGGTCACCTCAGGGCACTATGCAAATTGGTTTCAACAGAAACCCGGTCAAGCGCCTCGCGCACTTATCTTTGATACTGATAAGAAATATAGTTGGACGCCAGGTCGTTTCAGCGGTTCTCTGTTGGGCGCAAAAGCTGCGCTTACCATTAGCGATGCGCAACCTGAGGACGAGGCCGAGTACTATTGTTCGCTGTCGGATGTAGATGGATACCTATTTGGTGGCGGAACACAACTAACGGTGCTGTCCACCGGGCATCACCATCACCATCACTAA

1. ***Ptac-dL5-µNS***

***promoter-dL5-linker-µNS***

TGCACCAATGCTTCTGGCGTCAGGCAGCCATCGGAAGCTGTGGTATGGCTGTGCAGGTCGTAAATCACTGCATAATTCGTGTCGCTCAAGGCGCACTCCCGTTCTGGATAATGTTTTTTGCGCCGACATCATAACGGTTCTGGCAAATATTCTGAAATGAGCTGTTGACAATTAATCATCGGCTCGTATAATGTGTGGAATTGTGAGCGGATAACAATTTCACACAGGAAACAGCCTCGACAGGCCTAGGAATTCAATTAGGAGGTAATTAAGCATGCAGGCCGTAGTGACCCAAGAACCCAGTGTGACTGTCAGTCCAGGAGGCACCGTGATCTTAACTTGTGGTTCAGGTACTGGGGCGGTTACCAGCGGTCATTATGCTAATTGGTTTCAGCAAAAGCCAGGACAAGCTCCGCGCGCGCTTATTTTCGACACCGACAAGAAATACTCTTGGACTCCTGGGCGCTTTTCAGGCAGTTTACTGGGCGCGAAAGCGGCTTTGACGATCTCTGACGCACAGCCAGAGGATGAAGCGGAATATTACTGTAGCCTGTCTGACGTAGATGGCTATTTATTTGGCGGAGGTACTCAACTTACGGTGTTGTCCGGAGGCGGTGGGTCGGGCGGTGGGGGCTCCGGAGGTGGCGGAAGCGGAGGGGGAGGCAGCCAAGCAGTGGTTACTCAGGAGCCGTCTGTTACCGTGTCACCGGGCGGTACTGTGATTCTGACCTGCGGCTCGGGAACTGGGGCGGTCACCTCAGGGCACTATGCAAATTGGTTTCAACAGAAACCCGGTCAAGCGCCTCGCGCACTTATCTTTGATACTGATAAGAAATATAGTTGGACGCCAGGTCGTTTCAGCGGTTCTCTGTTGGGCGCAAAAGCTGCGCTTACCATTAGCGATGCGCAACCTGAGGACGAGGCCGAGTACTATTGTTCGCTGTCGGATGTAGATGGATACCTATTTGGTGGCGGAACACAACTAACGGTGCTGTCCACCGGGCATCACCATCACCATCACGGCGGTGGGGGCTTGTCTGCTGGCGCTGGGGGCGGAGGCAGCGCGTCTATGGTAGACGGGATTAAACTACAGTTGGACGCATCTAGACAGTGTCATGAGTGTCCCGTGTTGCAGCAGAAAGTAGTTGAGTTGGAAAAACAGATTATCATGCAGAAGTCCATTCAGTCAGATCCTACCCCAATGGCGCTGCAACCACTGTTATCTCAGTTGCGTGAACTGTCTAGTGAGGTCACCCGACTCCAGATGGAGTTAAGTCGGACTCAGTCCCTGAATGCTCAGTTGGAAGCGGATGCTAAGTCAGCTCAAGCATGTAGTCTGGATATGTATTTGAGACACCACACCTGCATTAATGGTCATACAAAAGAAGATGAACTGCTTGATGCTGTACGTGTCGCTCCAGATGTGAGGAAAGAAATCATGGAAAAGAGGGGCGAAGTGAGAAGGGGCTGGTGCGAACGTATCTCTAAGGAAGCGGCTGCCAAATGCCAAACTGTTATTGATGACTTGACTCAGATGAATGGAAAGCAGGCACGAGAGATAACAGAATTACGCGAGTCAGCCGAGAATTATGAGAAGCAGATTGCGGAATTGGTGGGCACTATTACTCAAAACCAGATGACGTATCAGCAAGAGCTACAAGCTTTGGTAGCGAAGAATGTGGAACTGGATACGATGAACCAACGTCAGGCTAAATCATTGCGTATTACTCCCTCCCTTCTATCAGCCACTCCTATCGATTCAGTCGACGGCGCTGCTGACCTGATTGATTTCTCCGTTCCAACTGATGAGCTGTAA
