# Supplementary material for: Biofilm dispersal patterns revealed using far-red fluorogenic probes
Source: PLoS Biol. 2024 Nov 25;22(11):e3002928. doi: 10.1371/journal.pbio.3002928 (PMC11627390; doi:10.1371/journal.pbio.3002928)
Supplement: S3 Table — List of synthetic DNA used in the study. (DOCX) [file pbio.3002928.s019.docx]

**S3 Table. Oligos and synthetic DNA used in this study.**

| Oligo # | Name | Binding location | Direction | 5' to 3' Sequence |
| --- | --- | --- | --- | --- |
| 105 | BBC1881 | Upstream vc_1807 | F | TTTAAAGGGGATCAGTGACCG |
| 106 | BBC1882 | Downstream vc_1807 | R | CAATTTTGCTTTTGGACCATCCC |
| 270 | 1807_2700up | Upstream vc_1807 | F | GGCCGGCACTTTGATTACAATC |
| 271 | 1807_2700down | Downstream vc_1807 | R | GTCTATATCAGAGCGCTTAAAGAGCG |
| 512 | Ptac_RBS_R | RBS used with Ptac | R | gcttaattacctcctaattgaattcctaggcctg |
| 232 | ABD123 | Universal upstream AbR cassette | F | ATTCCGGGGATCCGTCGAC |
| 1403 | Vc_mNeonGreen_dL5_Vc_opt | mNeonGreen-dL5 gBlock | - | CCTAGGAATTCAATTAGGAGGTAATTAAGCATGGTATCGAAGGGGGAGGAAGATAATATGGCTTCACTTCCAGCTACACACGAGCTTCACATTTTTGGTTCTATCAACGGAGTTGACTTTGACATGGTCGGACAAGGTACTGGCAATCCGAACGACGGTTACGAGGAATTAAATCTTAAATCTACGAAAGGGGACTTACAGTTTTCACCCTGGATCTTAGTGCCGCATATCGGTTACGGGTTCCACCAATATCTCCCGTACCCTGACGGAATGTCGCCTTTCCAGGCAGCTATGGTGGACGGTTCAGGGTATCAGGTACATCGTACCATGCAATTTGAAGATGGCGCATCTCTTACAGTGAACTATCGCTACACGTATGAAGGTAGTCACATCAAGGGTGAGGCCCAAGTAAAGGGGACAGGCTTCCCAGCTGACGGACCTGTTATGACAAATTCGTTGACAGCGGCAGATTGGTGCCGCTCTAAAAAGACTTACCCTAATGACAAGACTATTATCAGTACCTTCAAGTGGAGCTACACAACGGGTAACGGGAAACGCTACCGTTCAACGGCACGCACCACCTATACTTTCGCAAAACCGATGGCAGCCAACTACCTCAAGAATCAACCAATGTATGTATTCCGCAAAACCGAACTGAAGCACTCGAAGACCGAACTGAACTTTAAGGAATGGCAAAAAGCCTTTACAGACGTTATGGGGATGGATGAATTATATAAGGGCGGTGGGGGCTTGTCTGCTGGCGCTGGGGGCGGAGGCAGCGCGTCTCAGGCCGTAGTGACCCAAGAACCCAGTGTGACTGTCAGTCCAGGAGGCACCGTGATCTTAACTTGTGGTTCAGGTACTGGGGCGGTTACCAGCGGTCATTATGCTAATTGGTTTCAGCAAAAGCCAGGACAAGCTCCGCGCGCGCTTATTTTCGACACCGACAAGAAATACTCTTGGACTCCTGGGCGCTTTTCAGGCAGTTTACTGGGCGCGAAAGCGGCTTTGACGATCTCTGACGCACAGCCAGAGGATGAAGCGGAATATTACTGTAGCCTGTCTGACGTAGATGGCTATTTATTTGGCGGAGGTACTCAACTTACGGTGTTGTCCGGAGGCGGTGGGTCGGGCGGTGGGGGCTCCGGAGGTGGCGGAAGCGGAGGGGGAGGCAGCCAAGCAGTGGTTACTCAGGAGCCGTCTGTTACCGTGTCACCGGGCGGTACTGTGATTCTGACCTGCGGCTCGGGAACTGGGGCGGTCACCTCAGGGCACTATGCAAATTGGTTTCAACAGAAACCCGGTCAAGCGCCTCGCGCACTTATCTTTGATACTGATAAGAAATATAGTTGGACGCCAGGTCGTTTCAGCGGTTCTCTGTTGGGCGCAAAAGCTGCGCTTACCATTAGCGATGCGCAACCTGAGGACGAGGCCGAGTACTATTGTTCGCTGTCGGATGTAGATGGATACCTATTTGGTGGCGGAACACAACTAACGGTGCTGTCCACCGGGCATCACCATCACCATCACTAAGTGTGAATTCCGGGGATCCGTCGACCTGCAGTTCGA |
| 1402 | Vc1807_SSMBP_dL5_peri_Vc_opt | Secretion signal MBP-dL5 gBlock | - | CCTAGGAATTCAATTAGGAGGTAATTAAGCTTGGTGAGCCCTACAACACAAAAAGAAAAGGATATGAACATGAAAAATGCCCTAAGCACAGTCGCGCTGAGCACTCTGGTGGCTCTTGGTTCGTTTGGTGCCCATGCTGCTGAAGCGGAAGCATCTGCTGGCGCTGGGGGCGGAGGCAGCGCGTCTATGCAGGCCGTAGTGACCCAAGAACCCAGTGTGACTGTCAGTCCAGGAGGCACCGTGATCTTAACTTGTGGTTCAGGTACTGGGGCGGTTACCAGCGGTCATTATGCTAATTGGTTTCAGCAAAAGCCAGGACAAGCTCCGCGCGCGCTTATTTTCGACACCGACAAGAAATACTCTTGGACTCCTGGGCGCTTTTCAGGCAGTTTACTGGGCGCGAAAGCGGCTTTGACGATCTCTGACGCACAGCCAGAGGATGAAGCGGAATATTACTGTAGCCTGTCTGACGTAGATGGCTATTTATTTGGCGGAGGTACTCAACTTACGGTGTTGTCCGGAGGCGGTGGGTCGGGCGGTGGGGGCTCCGGAGGTGGCGGAAGCGGAGGGGGAGGCAGCCAAGCAGTGGTTACTCAGGAGCCGTCTGTTACCGTGTCACCGGGCGGTACTGTGATTCTGACCTGCGGCTCGGGAACTGGGGCGGTCACCTCAGGGCACTATGCAAATTGGTTTCAACAGAAACCCGGTCAAGCGCCTCGCGCACTTATCTTTGATACTGATAAGAAATATAGTTGGACGCCAGGTCGTTTCAGCGGTTCTCTGTTGGGCGCAAAAGCTGCGCTTACCATTAGCGATGCGCAACCTGAGGACGAGGCCGAGTACTATTGTTCGCTGTCGGATGTAGATGGATACCTATTTGGTGGCGGAACACAACTAACGGTGCTGTCCACCGGGCATCACCATCACCATCACTAAGTGTGAATTCCGGGGATCCGTCGACCTGCAGTTCGA |
| 1555 | Vc1807_N-Ter_dL5_138_linker YSD_Vc-opt_MuNS | Ptac-dL5-µNS gBlock | - | TGCATTGGTACAGAAGGCGGGTAGCTAAGGTGCACCAATGCTTCTGGCGTCAGGCAGCCATCGGAAGCTGTGGTATGGCTGTGCAGGTCGTAAATCACTGCATAATTCGTGTCGCTCAAGGCGCACTCCCGTTCTGGATAATGTTTTTTGCGCCGACATCATAACGGTTCTGGCAAATATTCTGAAATGAGCTGTTGACAATTAATCATCGGCTCGTATAATGTGTGGAATTGTGAGCGGATAACAATTTCACACAGGAAACAGCCTCGACAGGCCTAGGAATTCAATTAGGAGGTAATTAAGCATGCAGGCCGTAGTGACCCAAGAACCCAGTGTGACTGTCAGTCCAGGAGGCACCGTGATCTTAACTTGTGGTTCAGGTACTGGGGCGGTTACCAGCGGTCATTATGCTAATTGGTTTCAGCAAAAGCCAGGACAAGCTCCGCGCGCGCTTATTTTCGACACCGACAAGAAATACTCTTGGACTCCTGGGCGCTTTTCAGGCAGTTTACTGGGCGCGAAAGCGGCTTTGACGATCTCTGACGCACAGCCAGAGGATGAAGCGGAATATTACTGTAGCCTGTCTGACGTAGATGGCTATTTATTTGGCGGAGGTACTCAACTTACGGTGTTGTCCGGAGGCGGTGGGTCGGGCGGTGGGGGCTCCGGAGGTGGCGGAAGCGGAGGGGGAGGCAGCCAAGCAGTGGTTACTCAGGAGCCGTCTGTTACCGTGTCACCGGGCGGTACTGTGATTCTGACCTGCGGCTCGGGAACTGGGGCGGTCACCTCAGGGCACTATGCAAATTGGTTTCAACAGAAACCCGGTCAAGCGCCTCGCGCACTTATCTTTGATACTGATAAGAAATATAGTTGGACGCCAGGTCGTTTCAGCGGTTCTCTGTTGGGCGCAAAAGCTGCGCTTACCATTAGCGATGCGCAACCTGAGGACGAGGCCGAGTACTATTGTTCGCTGTCGGATGTAGATGGATACCTATTTGGTGGCGGAACACAACTAACGGTGCTGTCCACCGGGCATCACCATCACCATCACGGCGGTGGGGGCTTGTCTGCTGGCGCTGGGGGCGGAGGCAGCGCGTCTATGGTAGACGGGATTAAACTACAGTTGGACGCATCTAGACAGTGTCATGAGTGTCCCGTGTTGCAGCAGAAAGTAGTTGAGTTGGAAAAACAGATTATCATGCAGAAGTCCATTCAGTCAGATCCTACCCCAATGGCGCTGCAACCACTGTTATCTCAGTTGCGTGAACTGTCTAGTGAGGTCACCCGACTCCAGATGGAGTTAAGTCGGACTCAGTCCCTGAATGCTCAGTTGGAAGCGGATGCTAAGTCAGCTCAAGCATGTAGTCTGGATATGTATTTGAGACACCACACCTGCATTAATGGTCATACAAAAGAAGATGAACTGCTTGATGCTGTACGTGTCGCTCCAGATGTGAGGAAAGAAATCATGGAAAAGAGGGGCGAAGTGAGAAGGGGCTGGTGCGAACGTATCTCTAAGGAAGCGGCTGCCAAATGCCAAACTGTTATTGATGACTTGACTCAGATGAATGGAAAGCAGGCACGAGAGATAACAGAATTACGCGAGTCAGCCGAGAATTATGAGAAGCAGATTGCGGAATTGGTGGGCACTATTACTCAAAACCAGATGACGTATCAGCAAGAGCTACAAGCTTTGGTAGCGAAGAATGTGGAACTGGATACGATGAACCAACGTCAGGCTAAATCATTGCGTATTACTCCCTCCCTTCTATCAGCCACTCCTATCGATTCAGTCGACGGCGCTGCTGACCTGATTGATTTCTCCGTTCCAACTGATGAGCTGTAAATTCCGGGGATCCGTCGACCTGCAGTTCGA |
| 1529 | mNeonGreen_Y69G_R | mNeonGreen(Y69G) | R | GTACGGGAGATATTGGTGGAACCCGCCACCGATATGCGGCACTAAGATCCAG |
| 1530 | mNeonGreen_Y69G_F | mNeonGreen(Y69G) | F | CTGGATCTTAGTGCCGCATATCGGTGGCGGGTTCCACCAATATCTCCCGTAC |
| 1525 | pBBR1-Ptac-mNeonGreen-dL5_plasR | pBBR1-Ptac-mNeonGreen-dL5 gibson | R | ggctgcctgacgccagaagcattggtgcaattgcgttgcgctcactgcccgctttc |
| 1526 | pBBR1-Ptac-mNeonGreen-dL5_plasF | pBBR1-Ptac-mNeonGreen-dL5 gibson | F | CCACCGGGCATCACCATCACCATCACTAAgcgttaatattttgttaaaattcgcg |
| 1527 | pBBR1-Ptac-mNeonGreen-dL5_insertF | pBBR1-Ptac-mNeonGreen-dL5 gibson | F | gaaagcgggcagtgagcgcaacgcaattgcaccaatgcttctggcgtcaggcagcc |
| 1528 | pBBR1-Ptac-mNeonGreen-dL5_insertR | pBBR1-Ptac-mNeonGreen-dL5 gibson | R | cgcgaattttaacaaaatattaacgcTTAGTGATGGTGATGGTGATGCCCGGTGG |
