# Supplementary material for: Biofilm dispersal patterns revealed using far-red fluorogenic probes
Source: PLoS Biol. 2024 Nov 25;22(11):e3002928. doi: 10.1371/journal.pbio.3002928 (PMC11627390; doi:10.1371/journal.pbio.3002928)
Supplement: Attachment — Submitted filename: FAPdispersal_ResponsetoReviewers_Submitted.docx [file pbio.3002928.s020.docx]

Dear Dr. Hodge,

Thank you for considering our manuscript. We are pleased that the reviewers and editors found our work to be of interest. The reviewers kindly provided suggestions to improve our manuscript. We have revised the manuscript exactly as requested. Below are our point-by-point responses to the reviewers’ comments. Below, reviewer comments are in black text and our responses are in green text. References to line numbers in the revised manuscript are relevant when Track Changes are “off.” We hope the manuscript is now acceptable for publication in *PLOS Biology*. Please let us know if you need anything more.

Sincerely,

Drew Bridges

REVIEWS:

Reviewer #1: This manuscript reports the development of a novel fluorescent reporter technique that consists of a small protein, dl5, and one of several different fluoregenic dyes that only fluoresce when bound to dl5. This reporter method is then used to track biofilm growth and dispersal over time for WT Vibrio cholerae and a number of deletion mutant derivatives. The work documents the spatial pattern of biofilm dispersal in V. cholerae with very good quantitative detail, including the contributions of native biofilm structure (rbmA deletion), motility/motion reversal (cheY) and matrix depolymersae secretion (lapG, rbmB).

As a primarily methods development paper, this manuscript is very exciting and in my opinion should be seen by as many microscopy researchers as possible, as quickly as possible. There are at least two tremendous benefits of the dl5-MGe method described here: (1) it allows for clear imaging in the absence of oxygen, a major field-limiting problem with live-imaging conventional fluorescent proteins, (2) its excitation/emission maxima (636/664) should be compatible when co-imaging with the modern conventional far-red fluorescnet proteins (e.g. mKate2 - 588/633). When used appropriately, this means that people formerly limited to imaging 3-4 channels in one sample can now image 4-5. The range of applications for this method are immense, to say the least.

The biological results are a little more modest/descriptive but still very good and add to the still-limited literature on the mechanistic details of biofilm dispersal. This is an important topic that is severrely under-studied at the cellular spatial scale. I don't have much to comment on with these sections of the paper, but one point that didn't quite land in my opinion was the result regarding fluid channels opening up in biofilm clusters during the dispersal process. I don't see what the authors are referring to regarding channels in any of the images in the paper - are they just talking about the opened spaces between cells in biofilms from which some cells have dispersed? Calling this space "channels" makes a connection to the idea of fluid transport channels in macroscopic biofilms that seems inappropriate and potentially confusing to people who don't work on these sytems. I would advise the authors to change the language here, but this is more of a semantic issue
than a technical one.

We sincerely thank the referee for their enthusiastic review of our manuscript.

To reconcile their valid point that referring to dynamic regions of dispersing biofilms as “channels” could be confusing (given terminology already used in other contexts for biofilms), we have replaced this term with the phrase “dynamic regions” in the revised text. We have also further clarified the properties of these regions, in short, they are regions of the biofilms where cells exhibit rapid outward directional movement compared to neighboring regions. These changes are reflected on lines 18, 61, 203, 220-222, 224, 226, 228, 247-248, 297, 299, 317, 344, 351, 358, 373, 399, 583, 592, 595-596, 821-822 in the revised manuscript.

Reviewer #2 (Erin Gloag, signs review): This manuscript is submitted to PLOS Biology for consideration as a Methods and Resources article. The new methods and resources presented is the use of fluorogen-activating proteins as a labelling technology to analyse biofilm formation of V. cholerae. A strength of this manuscript is that authors clearly demonstrate the utility of this technology over conventional fluorescent proteins to image biofilms, but they also use this technology to analyse biofilm dispersal and the spatiotemporal kinetics and cell-cell interactions during this stage of biofilm development. This is a well-written and really interesting manuscript that I enjoyed reading. The data presentation is also unique and clearly presents complicated time-lapse data. My main comment would be to expand the discussion to include some limitations of the technology. My specific comments are below. Finally the time-lapse microscopy was a joy to watch.

We appreciate the reviewer’s positive view of our manuscript, and we thank them for their constructive feedback.

Major comments
1.      Can authors comment on using this technology to image polymicrobial biofilms? For example, can different bacteria be tagged with different FAP sequences that bind to unique fluorogens?

This is a great point. Yes, we do anticipate that this technology could be used to image multiple species simultaneously. In the current work, we optimized the FAP dL5 for bacterial cell labeling, but there are other FAP-fluorogen pairs that span the visible spectrum and that we will pursue as additional labels in the future. We have added the following paragraph to the discussion:

(Line 410): We anticipate that the FAP-fluorogen labeling approaches described here will enable research in diverse areas of microbiology. Specifically, the oxygen independence of FAP fluorescence combined with the far-red spectral properties of the dL5-MG complex could enable interrogation of microbiome biogeography in live animals in unprecedented detail. Moreover, distinct FAP-fluorogen pairs, beyond the dL5-MG complex used here, have been developed to span the visible spectrum.^19,20^ These probes could be used to tag proteins of interest, to label multiple organisms simultaneously, or to monitor gene expression in complex polymicrobial communities. Thus, we propose that extensions of the FAP technology employed in this work could enable a mechanistic understanding of microbial community development from the level of single species to the level of complex multi-species assemblages.

2.      Can authors comment on using this technology in in vivo animal models. Or would this technology only me amendable in vitro?

We do believe there is a high likelihood that our labeling approach will work in animal models. Moreover, the far-red spectra of the dL5-MG complexes could enable deeper imaging that is possible with conventional fluorescent proteins. We have pointed out the potential for *in vivo* applications in the revised lines given in response to point 1 (Line 410).

3.      Methods. Include number of technical and biological replicates for each assay

We have more clearly delineated sample sizes for all experiments in the figure legends and in the methods.

4.      Methods. What software was used to perform the imaging analysis. The imaging analysis and data presentation is a strength of the manuscript, and increased transparency in the methods would increase the utility for the biofilm field.

The image analysis performed in this study leveraged custom generated scripts in the Julia programming language as described in the Methods. All of our annotated code is available for download on GitHub or Zenodo. We have clarified our methods to include the following statement:

(Line 530): All image analysis procedures were performed using the Julia programming language (version 1.10.2).^44,45^

Minor comments
5.      For clarity, I would suggest including the statistics on the graphs, rather than just in the figure legend alone as this gets lost.

We have made the suggested change.

6.      Include error in Figures 2E and F.

We have made the suggested change.

7.      Figure 4B. It is difficult to discern between the blue (cheY) and green (rbmB) lines. I would suggest picking a more contrasting colour for one of them (pink, purple or red?)

We now use crimson for rbmB.
